# Reduction of Oxidative Stress in Chronic Kidney Disease Does Not Increase Circulating α-Klotho Concentrations

**DOI:** 10.1371/journal.pone.0144121

**Published:** 2016-01-25

**Authors:** Aaltje Y. Adema, Frans J. van Ittersum, Joost G. Hoenderop, Martin H. de Borst, Prabath W. Nanayakkara, Piet M. Ter Wee, Annemieke C. Heijboer, Marc G. Vervloet, NIGRAM consortium

**Affiliations:** 1 Department of Nephrology, VU University Medical Center, Amsterdam, The Netherlands; 2 Department of Physiology, Radboud university medical center Nijmegen, The Netherlands; 3 Department of Internal Medicine, Division of Nephrology, University Medical Center Groningen, Groningen, The Netherlands; 4 Department of Internal Medicine, VU University Medical Center, Amsterdam, The Netherlands; 5 Department of Clinical Chemistry, VU University Medical Center, Amsterdam, The Netherlands; 6 Institute for Cardiovascular Research VU (ICaR-VU), Amsterdam. The Netherlands; Universidade de São Paulo, BRAZIL

## Abstract

The CKD-associated decline in soluble α-Klotho levels is considered detrimental. Some i*n vitro* and *in vivo* animal studies have shown that anti-oxidant therapy can upregulate the expression of α-Klotho in the kidney. We examined the effect of anti-oxidant therapy on α-Klotho concentrations in a clinical cohort with mild tot moderate chronic kidney disease (CKD). We performed a post-hoc analysis of a prospective randomized trial involving 62 patients with mild to moderate CKD (the ATIC study), all using an angiotensin-converting enzyme inhibitor (ACEi) or angiotensin receptor blocker (ARB) for 12 months. On top of that, the intervention group received anti-oxidative therapy consisting of the combination of pravastatin (40 mg/d) and vitamin E (α-tocopherol acetate, 300 mg/d) while the placebo was not treated with anti-oxidants. α-Klotho concentrations were measured at baseline and after 12 months of anti-oxidant therapy. Data were analysed using T-tests and Generalized Estimating Equations, adjusting for potential confounders such as vitamin D, parathyroid hormone, fibroblast-growth-factor 23 (FGF23) and eGFR. The cohort existed of 62 patients with an eGFR (MDRD) of 35 ± 14 ml/min/1.72m^2^, 34 were male and mean age was 53.0 ± 12.5 years old. Anti-oxidative therapy did successfully reduce oxLDL and LDL concentrations (P <0.001). α-Klotho concentrations did not change in patients receiving either anti-oxidative therapy (476.9 ± 124.3 to 492.7 ± 126.3 pg/mL, P = 0.23) nor in those receiving placebo 483.2 ± 142.5 to 489.6 ± 120.3 pg/mL, P = 0.62). Changes in α-Klotho concentrations were not different between both groups (p = 0.62). No evidence was found that anti-oxidative therapy affected α-Klotho concentrations in patients with mild-moderate CKD.

## Introduction

Prevention and better treatment of cardiovascular (CV) disease in CKD is one of the main challenges in current nephrology. Despite successful interventions on traditional risk factors such as blood pressure, dyslipidaemia and proteinuria, CV risk in CKD remains excessively high [1]. A rapidly increasing body of evidence supports involvement of the fibroblast growth factor 23 (FGF23)-klotho-vitamin D axis in the pathogenesis of cardiovascular disease in CKD patients [2]. Therapeutic reduction of oxidative stress in patients with CKD, for instance with HMG-CoA reductase inhibitors, could improve CV outcome [3]. However, whether antioxidant therapy induces favorable changes in the FGF23-Klotho-vitamin D axis and thereby improvements in CV outcome is unknown.

The α-Klotho gene was discovered in 1997 as an anti-aging gene [4]. α-Klotho is predominantly produced in the kidneys [5]. α-Klotho expression is downregulated by CKD-associated uremic toxins and oxidative stress and enhanced activity of the renin-angiotensin-aldosterone system (RAAS) [6–8]. A vicious circle could ensue, because vitamin D deficiency increases renin and subsequently angiotensin II, which is known to suppress α-Klotho and leads to subsequent high FGF23 concentrations, which in turn suppress vitamin D activation[9]. Lower α-Klotho concentrations are associated with progressive CKD[10], higher prevalence of cardiovascular disease[11], arterial stiffness [12], vascular calcification[13].

Therefore, increasing α-Klotho could be a legitimate goal in CKD patients. Several recent studies assessed different experimental options to up-regulate endogenous α-Klotho [7,9,14–24] through therapeutic reduction of oxidative stress with the administration of anti-oxidants [9,21,22] and HMG-CoA reductase inhibitors [23,24] However, whether anti-oxidant therapy increases α-Klotho in patients is unknown.

We hypothesized that a 12-month anti-oxidant therapy compared to placebo, induces an increase of α-Klotho. Therefore, in this study we measured α-Klotho before and after treatment with anti-oxidative therapy (vitamin E combined with a statin) or placebo in patients with mild to moderate CKD who participated in the Anti-Oxidant Therapy in Chronic Renal Insufficiency (ATIC) Study.

## Methods and Materials

### Study population

For the current analysis we performed a post-hoc analysis on subjects from the prospective randomised ATIC trial of whom blood and urine samples were available (25). Originally in this study 93 subjects with mild-moderate CKD (creatinine clearance of 15–70 mL/min/1.73m^2^ according to the Cockroft-Gault equation), on background treatment of inhibition of the renin-angiotensin system (RAS) by either angiotensin receptor blockers (ARB) or angiotensin converting enzyme (ACE)-inhibition were randomised to either a anti-oxidant regime comprising of pravastatin (40 mg/d) starting at baseline followed by the addition of vitamin E (α-tocopherol acetate, 300 mg/d) at month 6, or placebo for 12 months. Endpoints of the original study were common carotid intima-media thickness, brachial artery flow-mediated dilatation, albuminuria and renal function (eGFR). Details and results of the study have been published previously [25]. Excluded were subjects with diabetes, active vasculitis, nephrotic syndrome, renal transplantation, a fasting cholesterol level higher than 270 mg/dL (7.00 mmol/L), cholesterol-lowering therapy within 3 months prior to inclusion, or ischemic coronary, cerebrovascular, or peripheral arterial disease. The patients included in the ATIC study already provided informed consent for their samples to be stored and used in the future for research purposes without any further asking.

### Data collection

Of the 93 patients from the ATIC study, complete serum samples were available from 62 patients, which formed the base of the current analysis. The following data were recorded: age, gender, body mass index, smoker or non-smoker, renal function as determined by eGFR (MDRD) and serum creatinine (μmol/L). Plasma-oxidized low-density lipoprotein (oxLDL) and malondialdehyde were measured as quantitative indicators of oxidative stress. α-Klotho and vitamin D (25(OH)D) and phosphate concentrations were measured in serum samples and parathyroid hormone (PTH) and c-terminal fibroblast-growth-factor-23 (cFGF23) concentrations in plasma samples, all at baseline and after 12 months of treatment. The α-Klotho immunoassay (IBL international GmbH, Hamburg, Germany) was used to measure α-Klotho [26] with an intra-assay variation of <5% and an inter assay variation < 7.5%; c-terminal FGF23 was measured in EDTA-plasma using ELISA (Immutopics) [27]with an intra-assay variation of <5% and an inter assay variation < 10%. 25(OH)D was measured using the radioimmunoassay (Diasorin) (intra-assay variation of <10% above a concentration of 25 nmol/L and inter assay variation < 11% above a concentration of 25 nmol/L) and PTH using an automated immunoassay (Architect Abbott Diagnostics) (both intra- and inter assay variation <5% above a concentration of 1 pmol/L). Creatinine and phosphate were measured in 24-hour urine samples. All assays were run in the year 2013.

### Statistical analysis

Continuous variables are given as mean ± standard deviation for normally distributed data or as median [interquartile range] for skewed data. Categorical variables are given as number of patients and percentages. Normally distributed numerical variables were compared using an unpaired T-test, otherwise Mann-Whitney U test was used; categorical variables were compared by Chi-square test. Changes within the two treatment groups of oxLDL, LDL, MDA, eGFR and cFGF23 were analysed using paired T-test; between group differences were analysed using an unpaired T-test on deltas (Δ). In order to adjust the effects of the two treatment strategies on α-Klotho for confounders, General Estimating Equations, a regression technique suitable for longitudinal data analysis, was used. In the model with α-Klotho as dependent variable and treatment as determinant, vitamin D, parathyroid hormone, cFGF23 and renal function were added as confounders. In addition, to evaluate effect modification of the intervention, the product of time and group (time*group) was added as an independent variable in these analysis. A non-parametric correlation analysis was made between α-Klotho levels and FGF23 using Spearman’s rho. P-values < 0.05 were considered statistically significant. All analyses were performed using IBM SPSS Statistics software version 20. Details of statistical analysis are available in the S1 File.

## Results

### Characteristics of the study population

Data were analysed from 62 patients, 34 patients in the anti-oxidant intervention group, 28 in placebo group. In our cohort were fewer men than woman but otherwise it is a representative reflection of the original ATIC cohort. The mean age of the participants was 53 years old (range from 20–79 years old) and all patients suffered from chronic kidney disease stage 3–4 (eGFR 15–60 mL/min/1.73 m^2^)[28]. At baseline, there were no important differences in demographic and clinical parameters between both arms, as shown in Tables 1 and 2. Full data set available in the S2 File.

**Table 1:** Baseline characteristics of participants.

	Placebo group (n = 28)	Intervention group (n = 34)
Age (years)	51 ± 15	54 ± 11
Male, no. (%)	18 (64.3)	16 (47.1)
BMI (kg/m2)	25.9 ± 4.0	26.5 ± 4.9
Smokers, no. (%)	12 (52.2)	11 (47.8)
eGFR (ml/min/1.73m2)	38 (15)	33 (13)
25(OH)D (nmol/L)	70.2 ± 31.0	66.9 ± 32.5
PTH (pmol/L) (median + IQR)	8.9 (5.2–13.1)	8.6 (4.9–13.0)

Values are expressed as mean ±SD, unless specified otherwise. IQR = interquartile range

**Table 2:** Time-related results within and between groups. Values are expressed as mean with 95% confidence interval (95%CI).

	PLACEBO (n = 28)			INTERVENTION (n = 34)			p for between-group difference
	*Baseline*	*After 12 months of treatment*		*Baseline*	*After 12 months of treatment*		
	Mean (95%CI)	Mean (95%CI)	p	Mean (95%CI)	Mean (95%CI)	p	
Phosphate (mmol/L)	1.19 (1.11–1.28)	1.29 (1.19–1.40)	0.01	1.27 (1.14–1.39)	1.35 (1.18–1.51)	0.26	0.07
oxLDL (U/L)	60.2 (53.9–66.5)	63.4 (56.6–70.1)	0.03	66.8 (62.1–71.4)	55.1 (62.1–71.4)	<0.001	<0.001
LDL (mmol/L)	3.2 (2.9–3.6)	3.4 (3.0–3.7)	0.22	3.8 (3.5–4.2)	2.6 (2.3–2.9)	<0.001	<0.001
MDA (μmol/L)	7.7 (7.1–8.2)	8.0 (7.6–8.5)	0.10	7.7 (7.2–8.1)	7.8 (7.4–8.2)	0.38	0.37
eGFR (mL/min/1.73m^2^)	38 (32–43)	37 (31–43)	0.30	33 (29–38)	32 (27–37)	0.10	0.60
cFGF23* (RU/mL)	150.0 (90.8–236.8)	145.0 (95.0–359.0)	0.001	181.0 (130.0–310.5)	179.0 (137.8–318.0)	0.14	0.33
α-Klotho (pg/mL)	483.2 (427.9–538.5)	489.6 (443.0–536.3)	0.62	476.9 (433.6–520.3)	492.7 (448.6–536.7)	0.23	0.62
Urinary phosphate (mmol/L)	12.3 (10.0–14.6)	12.4 (9.8–15.0)	0.97	11.6 (8.8–14.4)	9.3 (7.7–11.0)	0.02	0.32

* cFGF23 is expressed as median and interquartile range.

#### Markers of oxidative stress

After 12 months, there was a statistically significant reduction of oxLDL (from 66.8 U/L ± 13.3 to 55.2 U/L ± 12.9, P<0.001) and low-density lipoprotein cholesterol (from 3.8 ± 0.93 mmol/L to 2.6 ± 0.71mmol/L, P<0.001) levels within the intervention group. There was no reduction of plasma malondialdehyde (from 7.7 ± 1.3 μmol/L to 7.8 ± 1.1 μmol/L, P = 0.38) during the study period. Levels of oxLDL, low-density lipoprotein cholesterol and plasma malondialdehyde all increased in the placebo group (from 60.2 U/L ± 15.5 to 63.4 U/L ± 16.7, from 3.2 ± 0.8 mmol/L to 3.4 ± 0.8 mmol/L, and from 7.7 ± 1.3 μmol/L to 8.0 ± 1.2 μmol/L resp.). However, this increase was only significant in the oxLDL levels (see Table 2).

### Renal function and cFGF23

After 12 months, the mean eGFR had decreased from 33 ± 13 mL/min/1.73m^2^ to 32 ± 14 mL/min/1.73m^2^ in the intervention group (P = 0.10) and decreased from 38 ± 15 mL/min/1.73m^2^ to 37 ± 16 mL/min/1.73m^2^ in the placebo group (P = 0.30) (P = 0.60 for between-group difference).

The median of cFGF23 changed from 181.0 RU/mL (IQR: 130.0–310.5) to 179.0 RU/mL (137.8–318.0) in the intervention group (P = 0.14). In the placebo group the median of cFGF23 changed from 150.0 (90.8–236.8) RU/mL to 145.0 (IQR: 95.0–359.0) RU/mL (P = 0.001) (P for between-group difference = 0.33).

### Effect of anti-oxidative therapy on circulating Klotho concentrations

**Fig 1:**
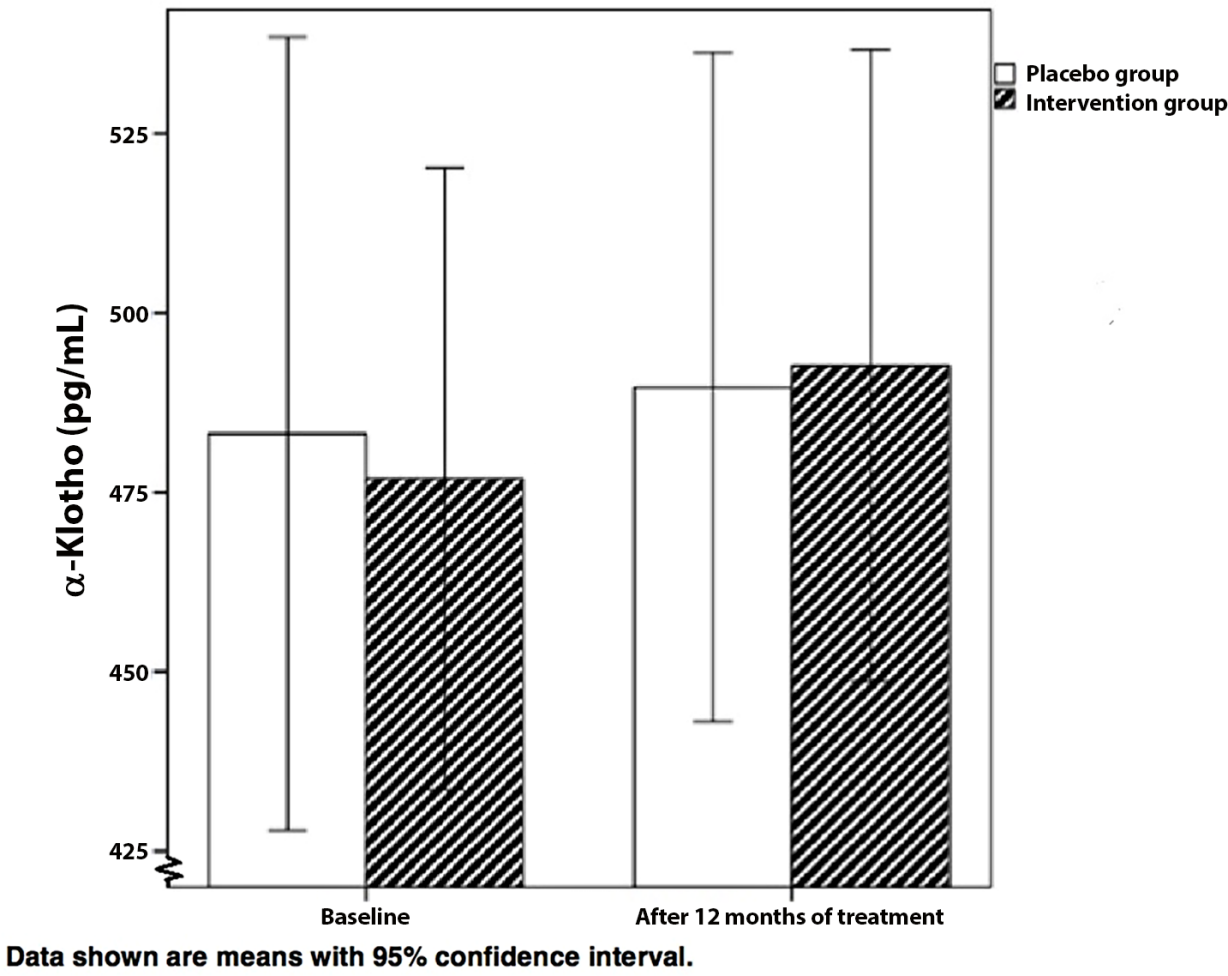
The effect of anti-oxidative therapy on α-Klotho concentrations. Data shown are means with 95% confidence interval.

α-Klotho concentrations at baseline increased from mean 476.9 ± 124.3 pg/mL to mean 492.7 ± 126.3 pg/mL for the intervention group (P = 0.23). In the placebo group α-Klotho concentrations increased from mean 483.2 ± 142.5 pg/mL to mean 489.6 ± 120.3 pg/mL (P = 0.62). The interaction term showed that α-Klotho levels did not significantly change over time in the intervention group compared to the placebo group (P = 0.62, Fig 1, and model 2, Table 3). Adjusting for the potential confounders eGFR, cFGF23, PTH and 25 hydroxyvitamin D3 did not influence results between the groups (p = 0.89, model 3 in Table 3).

**Table 3:** The effect of anti-oxidant treatment on α-Klotho in a multivariable GEE analysis.

Intervention versus placebo (change in pg/ml α-Klotho)	β (95% CI)
Crude	-1.6 (-62.5–59.3)
Model 1^1^	8.4 (-53.8–70.6)
Model 2^2^	3.6 (-62.8–70.0)
Model 3^3^	8.9 (-71.5–89.4)

^1^ Model 1: Adjusted for time course, MDRD

^2^ Model 2: Adjusted for variables from Model 1, plus an interaction term between group and time

^3^ Model 3: Adjusted for all variables from Model 2 plus 25OH-vitamin D, cFGF23, PTH, phosphate, calcium

We found no correlation between α-Klotho concentrations and cFGF23 levels (Spearman correlation coefficient = -0.045, p = 0.73).

## Discussion

This study demonstrates that there is no effect of the combined treatment with pravastatin and vitamin E as anti-oxidative therapy on α-Klotho concentrations in patients with mild-moderate CKD, despite that efficacy of anti-oxidative therapy to reduce oxidative stress was demonstrated by a decline of oxLDL concentrations.

Our data contradict experimental in vitro and in vivo animal studies that showed decreased Klotho expression in the kidney following oxidative stress. Subsequent administration of a free radical scavenger prevented this Klotho downregulation by oxidative stress [7,9,21,22,29–31]. The lack of an increase in α-Klotho concentrations under clinical anti-oxidative therapy in our study could have several explanations. First of all, we measured circulating α-Klotho concentrations; most of the previous in vitro and in vivo animal studies measured renal membrane bound Klotho. Second, we do not know if the anti-oxidative therapy patients received in this study was of sufficient potency to induce an upregulation of α-Klotho. Although the decline in oxLDL was statistically significant, we cannot exclude that more pronounced attenuation of oxidative stress would have resulted in higher concentrations of α-Klotho. However, Tamura et al. treated hypercholesterolemic patients during 8 weeks with a lower dose pravastatin than in our study (10 mg/day vs 40 mg/day) and they showed a significant reduction in plasma malondialdehyde with 14±13% [32]. Administration of rosuvastatin 20mg/day reduced both oxLDL and malondialdehyde levels in patients with atherosclerotic cerebrovascular disease [33]. Supplementation of vitamin E also has shown to decrease oxidative stress in an earlier study in patients with end-stage kidney disease [34]. We were not informed about therapy compliance of the patients in the ATIC study. However, given the response in both LDL cholesterol and oxLDL this is an unlikely explanation for the lack of effects on α-Klotho.

Even more, the patients in this study all used an ACEi or ARB from at least 2 weeks before baseline. Karalliedde et al. showed that treatment with valsartan, an ARB, is associated with an increase in sKlotho levels in patients with type 2 diabetes, systolic hypertension and albuminuria [35]. It is therefore conceivable that an previous increment of α-Klotho concentrations induced by inhibitors of the RAS prevented a subsequent further rise by our anti-oxidant strategy. The patients in our study had indeed higher baseline concentrations of α-Klotho when compared to the α-Klotho concentrations in patients with CKD stage 3 and 4 mentioned in the study of Kim et al. (10)

No data on α-tocopherol concentrations are available for our study, therefore we cannot exclude that α-tocopherol concentrations were sufficient at baseline, limiting an effect of anti-oxidative therapy on α-Klotho concentrations.

There are some limitations to this study that need to be acknowledged. The sample size of 62 patients is relatively small, leaving the possibility of a type II error. This also limited the ability to correct for confounding. However, a statistically significant difference in oxidative stress, as measured by oxLDL, was found between the two treatment arms, implicating that a difference in α-Klotho would had been detected in case oxidative stress had been an important factor. We cannot rule out however that the magnitude of reduction in oxidative stress was insufficient to influence α-Klotho concentrations. In the original ATIC study however, the multistep intervention was sufficient to modulate common carotid intima media thickness, albuminuria and brachial artery flow-mediated dilation. In addition to the potential of a type II error, small sample size in general limits the external validity of our findings. Another potential weakness, as mentioned, is that all patients were using either ARB or ACE-inhibitors, which may have induced an unnoticed, increase in α-Klotho, and precluded a further increment.

Finally, the IBL-assay used to measure α-Klotho concentrations has also some shortcomings. It is uncertain if and which forms of α-Klotho are detected and a cross reactivity with other solutes cannot be excluded [26,36].

The strengths of this study are the prospectively collected data with a randomised design, and the attained contrast between treatment groups in terms of biochemical indices of oxidative stress.

In conclusion, this study could not confirm our hypothesis that anti-oxidative therapy with pravastatin and vitamin E increases α-Klotho concentrations in patients with mild-moderate CKD on background therapy with ACE-inhibitors. Further research on this topic is warranted.

## Supporting Information

S1 File (Final Statistical analysis ATIC-study PLOSone). (SAV)

S2 File (Final full data-base ATIC-study PLOSone). (SAV)
